# Symptomatic Cerebellar Ptosis After Wide Foramen Magnum Decompression for Chiari I Malformation

**DOI:** 10.7759/cureus.35635

**Published:** 2023-03-01

**Authors:** Anagha Shankar, Mishal Johny, Priya Vadekkatt Sambhukumar, Shankar Ayyappan Kutty

**Affiliations:** 1 Anatomical Sciences, Humanitas University, Milan, ITA; 2 Neurosciences, Meitra Hospital, Kozhikode, IND; 3 Anesthesiology, NMC Specialty Hospital, Abu Dhabi, ARE; 4 Neurosciences, NMC Specialty Hospital, Abu Dhabi, ARE

**Keywords:** surgical complication, cranioplasty, posterior fossa decompression, chiari malformation, cerebellar slump, cerebellar ptosis

## Abstract

We present the case of a 22-year-old who developed severe neck pain within two weeks after undergoing posterior fossa decompression for a symptomatic Chiari I malformation. A diagnosis of cerebellar ptosis was made after magnetic resonance imaging (MRI) and he underwent a partial cranioplasty, following which his symptoms resolved. The pathology, diagnostic criteria, and management options are discussed.

## Introduction

Suboccipital craniectomy with or without duraplasty has been the standard treatment for Chiari I malformations with syringomyelia in most centers around the world. While many of the common complications associated with this surgery, such as the formation of a pseudo-meningocele and nuchal tethering are fairly well known, cerebellar ptosis or cerebellar slump has received much less attention despite it being a severely disabling condition, with only a few scattered reports in the literature [1]. We report a patient who presented to us one month after a posterior fossa decompression for Chiari I malformation, with features suggestive of meningeal irritation, low intracranial pressure symptoms, or possible recurrent tonsillar herniation manifesting as recurrence of symptoms, with neck pain and headaches, which had started two weeks prior to presentation.

## Case presentation

**Figure 1:**
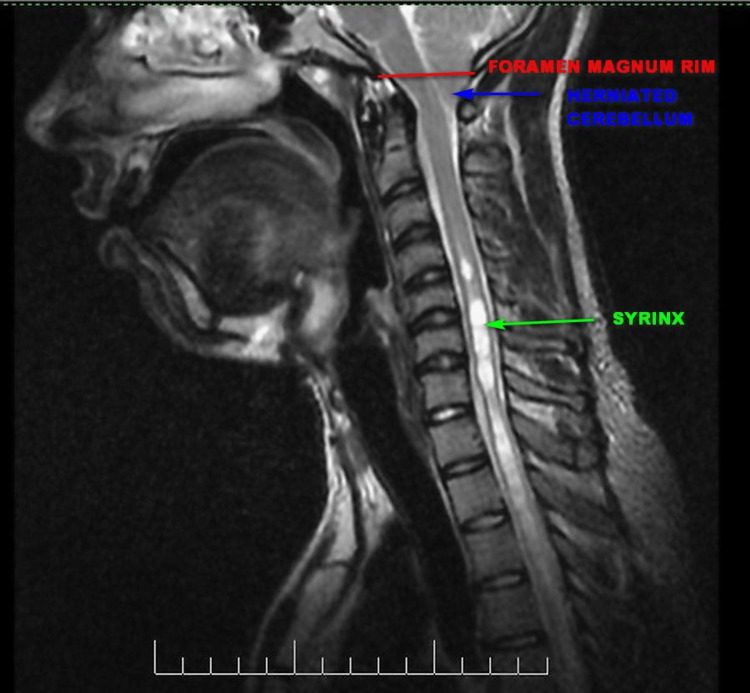
T2 weighted sagittal MRI scan done prior to the first surgery MRI: magnetic resonance imaging

A 22-year-old male, who had undergone foramen magnum decompression and duraplasty at another center for Chiari I malformation with syringomyelia (Figure 1), presented to us one month later, with severe headache and neck stiffness which had started two weeks after the surgery. After a lumbar puncture done at the initial treating hospital had ruled out bacterial meningitis, he had been treated with non-steroidal anti-inflammatory agents and then received oral dexamethasone for five days, before being sent to our center. The pain was made worse by extending the neck. There was no fever and no other symptoms. The dissociative anesthesia that had been his initial presenting symptom was persisting.

**Figure 2:**
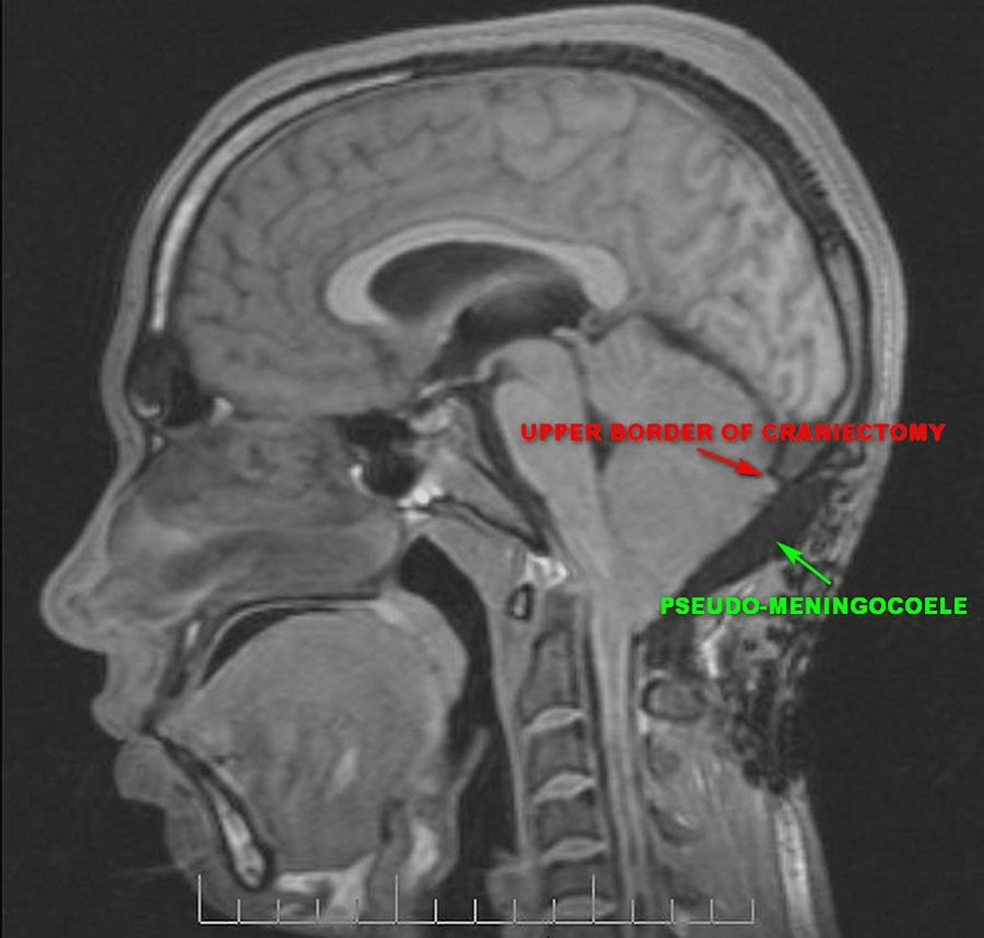
T1 T1 weighted sagittal MRI after the first surgery showing the extent of bone removal, the disappearance of the cervicothoracic syrinx, and the widening of the prepontine cistern. MRI: magnetic resonance imaging

A repeat cerebrospinal fluid analysis was within normal limits (glucose 48mg%, protein 55mg%, and white blood cell count of 12 cells per cu. mm. Staining for bacteria including tuberculosis and fungi was negative and aerobic culture yielded no growth). A magnetic resonance imaging (MRI) scan of the neck was done (Figure 2), which showed that the craniectomy defect extended up to the level of the tentorium cerebelli, with herniation of the cerebellum posteriorly and inferiorly. The cerebellar tonsils were at the level of the mid-body of the C2 vertebra, the cortical subarachnoid spaces posterior to the cerebellum were obliterated and a pseudo-meningocele was present at the operative site.

He underwent a partial suboccipital cranioplasty two months after the initial surgery. Bone cement was used to fashion a rectangular plate to reduce the size of the craniectomy to about 1.5cm from the foramen magnum. The cranioplasty plate was fixed to the lower end of the craniectomy using titanium plates, which were embedded into the cement before it set, and screws. Dura was not opened during the surgery, but a small defect that was seen along the edge of the previous duraplasty was repaired. The patient had significant relief from pain immediately after the surgery and was able to go back to work two weeks later. During the two years he was followed up, there was no recurrence of pain and his sensory symptoms had ameliorated.

## Discussion

Cerebellar ptosis was first reported by Williams in 1978 [2]. It is believed to result from the removal of too much bone during the craniectomy, resulting in loss of support to the cerebellum and its descent into a craniectomy defect. The pain associated with the condition is attributed to stretching of the dura, which has an abundance of nociceptive receptors [3]. Holly and Batzdorf [4] reported a series of seven patients with cerebellar ptosis, for whom three different surgical approaches were tried, namely, ventriculoperitoneal shunt, subdural peritoneal shunt, and methyl methacrylate bone cement reconstruction of the posterior fossa. The last method was the only one to produce symptomatic relief for the patients. More recently, studies have shown the efficacy of titanium mesh cranioplasty in achieving the same result [5].

**Figure 3:**
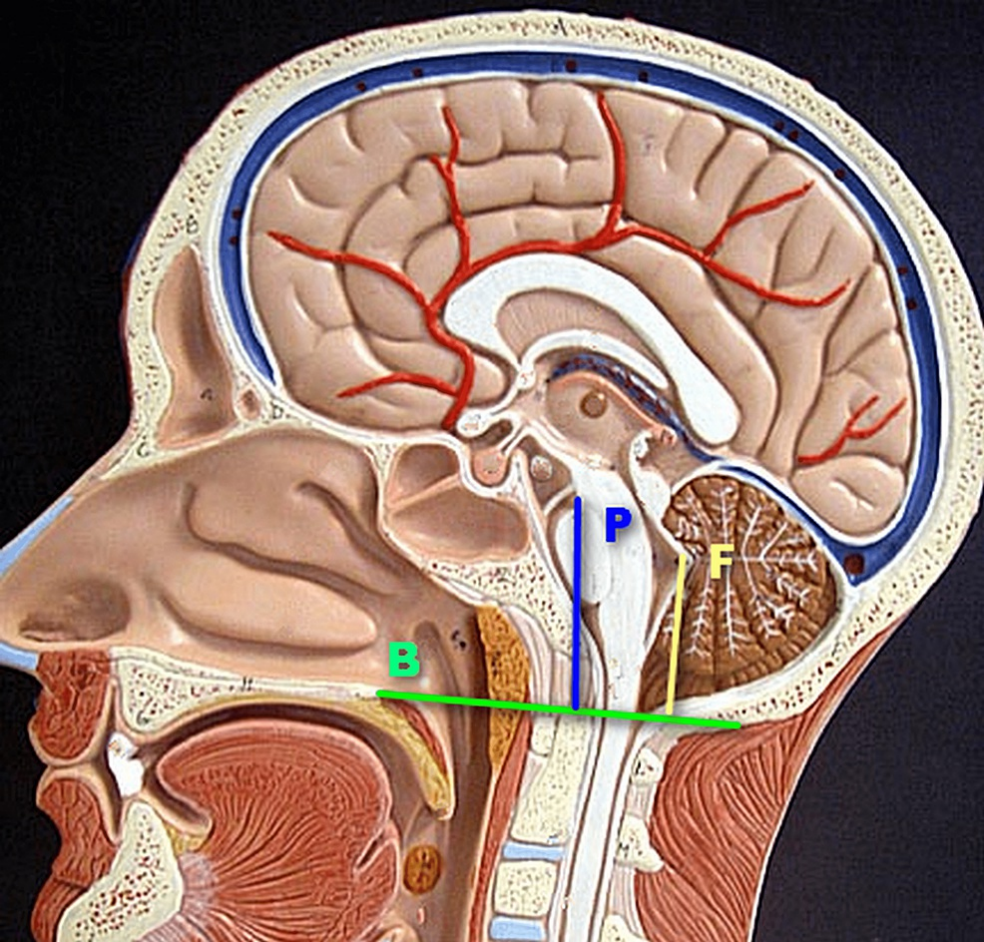
Lines drawn to diagnose cerebellar ptosis. B (green) is the baseline drawn along the line of the hard palate. P (blue) is the line from the baseline to the upper pons and F (yellow) is the line drawn from the baseline to the fastigium.

MRI scan is the investigation of choice for the diagnosis of cerebellar ptosis. On sagittal images (Figure 3), B is a line drawn along the hard palate to form the baseline, from which the height of the fastigium (F) and upper pons (P) is measured in millimeters. The values derived from the post-operative scan are subtracted from those derived from the pre-operative scan, and a difference of more than 2mm suggests a cerebellar slump [6]. The fastigium is used as it is a part of the cerebellum that is easily identifiable on sagittal MRI images, as the cerebellar tonsils may sometimes be resected during surgery and hence it may not be possible to assess the cerebellar descent in the post-operative scans by looking at the extent of cerebellum below the foramen magnum.

The extent of craniectomy and removal of foramen magnum rim should be governed by the amount of tonsillar or cerebellar herniation, and as a rule of thumb does not exceed 2.5cm [7]. However, this is only a rough guideline and needs to be personalized for each individual patient depending on the size of the posterior fossa. In the present case, the height of the posterior fossa was only 5.5cm and hence the size of the craniectomy was reduced to 1.5cm during the revision surgery. Various alternative techniques have been reported in the literature by different authors, which attempt to prevent the occurrence of cerebellar ptosis. These include expansile suboccipital cranioplasty, stealth cranioplasty, expansile cranioplasty with mesh-assisted dural tenting, and decompressive techniques such as "barrel staving" [8,9].

Most patients present several months to years after the initial surgery, with severe pain in the head and neck, radiating to the frontal region and jaw [1,10]. The pain is typically worse in coughing or straining. The differential diagnosis is nuchal tethering, where the dura becomes adherent to the overlying soft tissues and causes severe pain in neck movements. Our patient was unique in that his symptoms started within two weeks of surgery, and he presented one month after surgery. However, the prompt response to a partial cranioplasty proved the diagnosis.

## Conclusions

Utmost care must be maintained while performing a craniovertebral junction decompression for Chiari I, limiting bone removal to the bare minimum necessary for the individual patient. In our experience, excessive bone removal during posterior fossa decompression usually results from the use of a perforator to create a burr hole as the first step of the craniectomy. Due to the shape of the skull and the positioning of the head during the surgery, the burr hole is almost invariably made farther away from the foramen magnum rim than ideal, resulting in a large craniectomy. We would recommend using a surgical marker to draw out the intended area of bone removal on the skull, after exposure, and then using a high-speed drill to thin the bone out prior to removing it with a Kerrison rongeur. Alternative techniques such as posterior cranial fossa box expansion can be considered in difficult cases. Finally, the possibility of cerebellar ptosis must be considered even in patients who present with severe pain soon after surgery as it need not always be a long-term complication.
